# Extortion can outperform generosity in the iterated prisoner's dilemma

**DOI:** 10.1038/ncomms11125

**Published:** 2016-04-12

**Authors:** Zhijian Wang, Yanran Zhou, Jaimie W. Lien, Jie Zheng, Bin Xu

**Affiliations:** 1 Experimental Social Science Laboratory, Zhejiang University, Room 312, Dongsan Building, No. 866 Yuhangtang Road, Hangzhou 310058, China; 2 College of Economics, Zhejiang University, Room 326, Waijingmao Building, No. 38 Zheda Road, Hangzhou 310027, China; 3 Department of Decision Sciences and Managerial Economics, The Chinese University of Hong Kong, Cheng Yu Tung Building, No. 12 Chak Cheung Street, Hong Kong 999077, China; 4 School of Economics and Management, Tsinghua University, Room 561, Weilun Building, Haidian District, Beijing 100084, China; 5 Department of Economics, Esther Lee Building, Room 1013, The Chinese University of Hong Kong, Shatin, Hong Kong 999077, China; 6 College of Economics, Zhejiang Gongshang University, Economics Building, No. 18 Xuezheng Street, Hangzhou 310018, China

## Abstract

Zero-determinant (ZD) strategies, as discovered by Press and Dyson, can enforce a linear relationship between a pair of players' scores in the iterated prisoner's dilemma. Particularly, the extortionate ZD strategies can enforce and exploit cooperation, providing a player with a score advantage, and consequently higher scores than those from either mutual cooperation or generous ZD strategies. In laboratory experiments in which human subjects were paired with computer co-players, we demonstrate that both the generous and the extortionate ZD strategies indeed enforce a unilateral control of the reward. When the experimental setting is sufficiently long and the computerized nature of the opponent is known to human subjects, the extortionate strategy outperforms the generous strategy. Human subjects' cooperation rates when playing against extortionate and generous ZD strategies are similar after learning has occurred. More than half of extortionate strategists finally obtain an average score higher than that from mutual cooperation.

## Abstract (web summary)

The zero-determinant (ZD) strategies discovered by Press and Dyson overturned several decades of consensus about the iterated prisoner's dilemma. Here, the authors provide the first empirical evidence in support of Press and Dyson's theory, by showing that knowledge of the opponent and the length of the interaction can facilitate the Generous and Extortionate ZD strategies as predicted.

## Graphical abstract

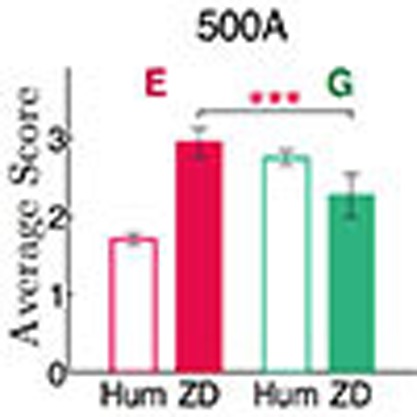

Promoting cooperation under adverse short-term individual incentives is an important social challenge, and the iterated prisoner's dilemma (IPD) has been widely studied as the canonical game theoretic framework representing this issue^1^,^2^,^3^,^4^,^5^,^6^,^7^. In a one-shot two-person prisoner's dilemma, there are two pure strategies: cooperate and defect. Each player receives *R* if they mutually cooperate; each player receives *P* if they mutually defect; if one player cooperates and the other defects, the defector receives *T* and the cooperator receives *S*, where *T*>*R*>*P*>*S* guarantees that in this game the commonly used solution concept Nash equilibrium is mutual defection, while 2*R*>*T*+*S* implies that mutual cooperation is actually the socially best outcome.

Ever since the computerized tournaments conducted by Axelrod^3^,^4^, kindness and fairness^8^,^9^,^10^ appeared to yield the best chance to promote and sustain cooperation. A substantial body of literature suggests that reciprocity makes mutual cooperation feasible^11^,^12^,^13^,^14^,^15^,^16^, and is a favourable strategy in an evolutionary setting^17^,^18^,^19^,^20^.

Recently, Press and Dyson^21^ discovered a surprising class of so-called zero-determinant (ZD) strategies which allow a player to unilaterally enforce a linear relationship between his score and that of his opponent. A ZD strategy is described by the probabilities of cooperation given the four possible outcomes of the previous round: *p*=(*p*_1_, *p*_2_, *p*_3_, *p*_4_), where *p*_*i*_, *i*∈(1, 2, 3, 4) is the probability of cooperation given the previous outcomes CC, CD, DC and DD, respectively. The premise is that by implementing particular randomizations of actions in a given period which are conditional on the previous period's outcome, the ZD strategist can induce an evolutionary opponent to always choose cooperation as the optimal strategy. A subclass of ZD strategies, namely the extortionate strategies, can further guarantee that the extortioner's own surplus exceeds the opponent's surplus by a fixed percentage. This means that the extortionate ZD strategist can maintain cooperation and pursue self-interest at same time. In addition, an extortioner can earn a score which exceeds the best possible score from another subclass of ZD strategies, generous ZD strategies. This new finding by Press and Dyson^21^ has stimulated many researchers to further investigate the performance of ZD strategies in various situations^22^,^23^,^24^,^25^,^26^,^27^,^28^,^29^,^30^,^31^. A key insight of the Press–Dyson theory^21^ is that if a human being's behaviour is developed in an evolutionary manner under an opponent's ZD strategy, he will tend to become more cooperative over time, and if so, the ZD strategist can achieve his maximum possible score by exploiting this cooperative tendency of his opponent^21^.

In spite of these significant developments in the literature, the empirical verification of the theory has been nontrivial. Until now, to our knowledge, there is just one published study which tests the Press–Dyson theory^21^ in a laboratory experiment. Hilbe *et al*.^26^ provided experimental evidence on the performances of different ZD strategies played by computers against human subjects. They specified the ZD strategists to play an extortionate strategy or a generous strategy against human subjects in the context of IPD, in which the extortionate strategy is predicted to earn a higher score based on the Press and Dyson's theory^21^. However, they find that generosity is in fact the more profitable strategy, and furthermore, that the cooperation rate of human co-players against extortionate strategies is only half of that against generous strategies. In other words, in practice, generosity appears to be the winning strategy after all. The experimental results by Hilbe *et al*.^26^ appear potentially at odds with the Press–Dyson prediction^21^, and have inspired other related studies^32^,^33^,^34^. This apparent inconsistency between theory and experimental results calls for a closer examination of the source of the discrepancy^35^. In the experiment by Hilbe *et al*.^26^, it was not made known to the human subjects that they were actually playing against a strategy executed by a computer program. Thus, in their experiment^26^, the effects of two factors, the effectiveness of ZD strategies themselves and human subjects' perception of the other player, may both be potentially responsible for the result they obtain. Another factor that may have led to the inconsistency between the Press–Dyson theory^21^ and the experimental result in Hilbe *et al*.^26^, is insufficient learning opportunities for the human subjects in their 60-round experiments. Until now, there has been no empirical evidence to illustrate the reality of the Press–Dyson theory^21^.

Our study attempts to establish an understanding and empirical validation of the Press–Dyson result^21^. We test the performance of the ZD strategies in a laboratory experiment of IPD which modifies the design of Hilbe *et al*.^26^, by introducing two factors: the knowledge of the opponent's computerized nature and the length of interaction. Each of these factors varies by two conditions, awareness versus unawareness by human subjects of the computerized nature of the opponent for the knowledge of the opponent's nature, and long rounds (500) versus short rounds (60) for length of interaction. Among these conditions, the treatments in which human subjects were both aware of the opponent's computerized nature as well as afforded rich learning experience over long-term interactions, was the ideal condition where the predictions of the Press–Dyson model^21^ could be realized. The remaining three settings further confirm the conditions (knowledge, length or both) that are necessary to produce the result. Our experiments show that under long rounds (500) and awareness conditions, the extortionate ZD strategy indeed outperforms the generous ZD strategy by inducing a higher cooperation rate among human co-players than under conditions of short rounds (60) or unawareness, and thus obtains a higher score by exploiting this tendency. Furthermore, under the awareness condition, after learning has occurred, the number of extortionate strategists who obtain an average score higher than that from mutual cooperation is significantly greater than the number of strategists who obtain an average score less than or equal to the score from mutual cooperation. To our knowledge, this is the first experimental evidence which supports the predictions of the Press–Dyson theory^21^.

## Results

### Experiment

**Figure 1:**
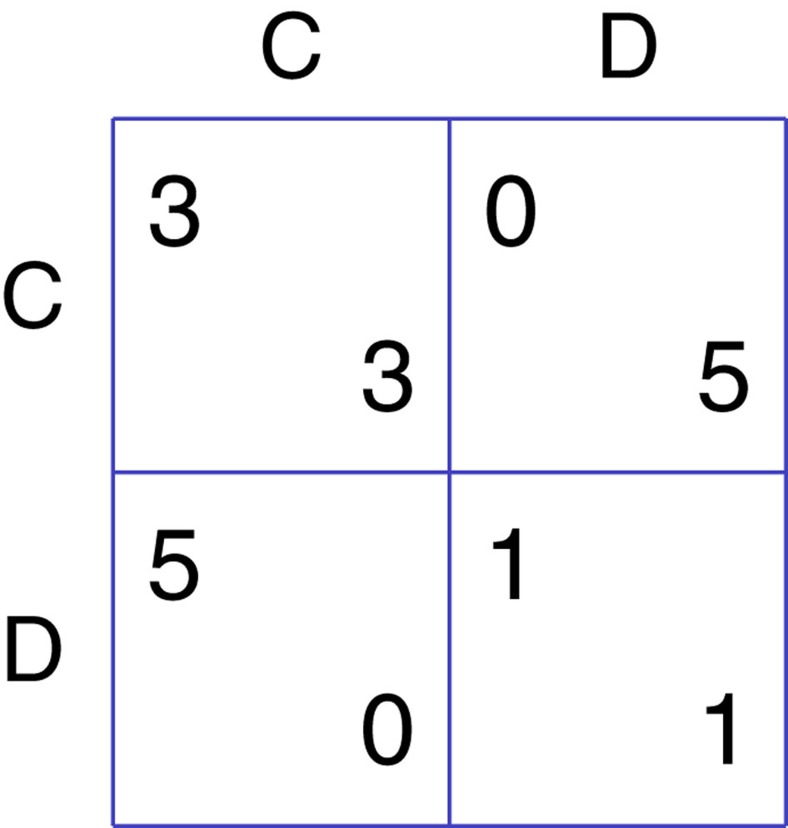
Payoff matrix. If both players cooperate, each player receives 3, if one cooperates and one defects, cooperator receives 0 and defector receives 5, if both defect, each player receives 1.

The IPD payoff matrix used in our experiment is shown in Fig. 1, which is identical to the one used in refs 21, 26. Both players receive 3 if they mutually cooperate, both players receive 1 if they mutually defect, the defector receives 5 and the cooperator receives 0 if one player cooperates and the other defects.

We implemented a set of treatments in which human subjects faced the computerized extortionate ZD strategy or generous ZD strategy, which further varied by the two additional aforementioned features: awareness versus unawareness by human subjects of the computerized nature of the opponent, and length of interaction (500 rounds versus 60 rounds). Table 1 summarizes the experimental design. For extortionate ZD strategy, the four conditional cooperation probabilities are (*p*_1_, *p*_2_, *p*_3_, *p*_4_)=(0.692, 0.000, 0.538, 0.000), and for generous ZD strategy the probabilities are (*p*_1_, *p*_2_, *p*_3_, *p*_4_)=(1.000, 0.182, 1.000, 0.364), where *p*_*i*_, *i*∈(1, 2, 3, 4) is the probability of cooperation given the previous outcome CC, CD, DC and DD, respectively. In addition, the extortionate ZD strategy defects in the first round and generous ZD strategy cooperates in the first round. These are the same conditional cooperation probabilities as the strong extortionate ZD strategy and strong generous ZD strategy in Hilbe *et al*.^26^. Theoretically, as proved by Press and Dyson^21^ and specified by Hilbe *et al*.^26^, for extortion, the ZD strategist's score 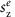 and his human co-player's score 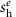 satisfy 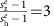, the maximum scores for the ZD strategist and the human co-player are 3.727 and 1.907, respectively. For generosity, the ZD strategist's score 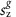 and his human co-player's score 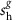 satisfy 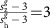, the maximum scores for both the ZD strategist and the human co-player are 3 (see Fig. 4). Therefore, if both types of ZD strategies can in fact induce human co-players' cooperative behaviour, the extortionate ZD strategists can achieve higher scores than the generous ZD strategists.

Altogether, 256 graduate and undergraduate students participated in the experiment with each treatment consisting of 32 participants. For further details on the implementation of the experiment, see the ‘Methods' section.

### Score performance of extortionate and generous ZD strategies

**Figure 2:**
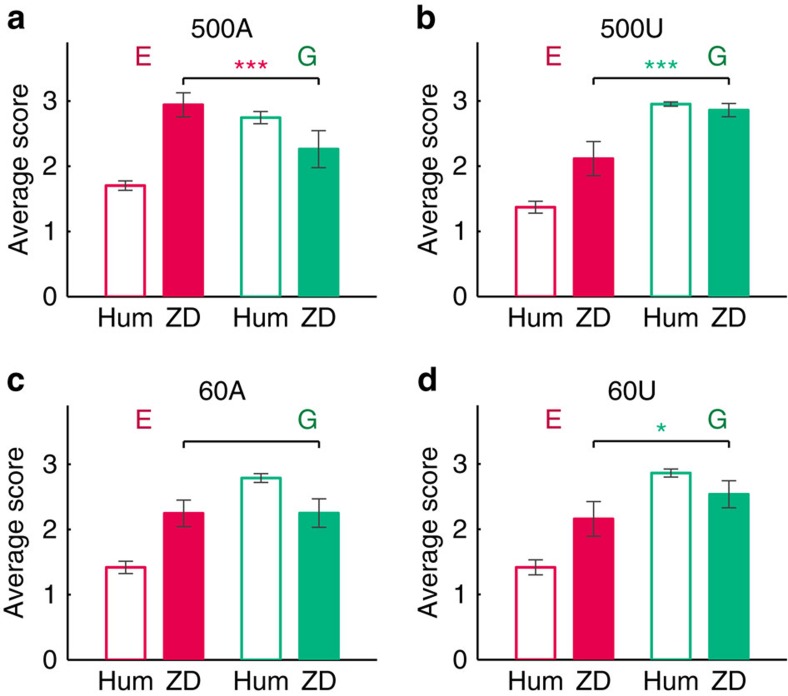
Average scores for human subjects and the ZD strategies for all treatments. Each graph shows the average score earned by human subjects (unfilled bars) and ZD strategists (filled bars), for the extortionate (red) and generous (green) treatments, respectively. The scores for the extortionate strategy are significantly higher than the generous strategy in treatment 500A (**a**), but not in the other treatments (**b**,**c**,**d**). The scores of the generous strategy are higher than the extortionate strategy in both of the unawareness treatments (**b**,**d**). There is no significant difference in scores between extortionate strategy and generous strategy in the 60A treatment (**c**). Three stars indicate *P*<0.001 (specifically, *P*=0.000 for the 500A treatment and *P*=0.000 for the 500U treatment), and one star indicates *P*<0.05 (specifically, *P*=0.019 for the 60U treatment). The error bars indicate the 95% confidence interval. For details on average scores and statistical results, see Supplementary Tables 1 and 2, respectively. For individual level data, see Supplementary Tables 3 and 4.

Figure 2 shows the resulting average scores over each treatment. To begin, we note the higher score achieved by the extortionate ZD strategist compared with the generous ZD strategist in the 500-round awareness treatments (500A) (Mann–Whitney's test, *n*_E_=*n*_G_=32, *z*=4.196, *P*=0.000), in which human subjects were aware of the computerized nature of the opponent. On the other hand, the corresponding 60-round awareness treatments (60A) yielded statistically identical average scores between the extortionate and generous strategists. Turning to the treatments in which human subjects were unaware of the computerized nature of the opponent, we note that the result of Hilbe *et al*.^26^ which demonstrates the favorability of the generous strategy, is replicated for the 60-round unawareness treatments (60U) and is even further enhanced in the 500-round unawareness treatments (500U). This indicates that neither awareness alone, nor long-term interaction alone, can deliver the prediction of Press–Dyson^21^. Rather, it is the combination of these features which creates the environment favourable to the extortionate ZD strategist.

In the treatment 500A, where support for the theory is clear, on average, the extortionate strategists earn 2.943±0.511 (mean±s.d.) scores per round and generous strategists earn 2.263±0.788 (mean±s.d.) scores per round. The average scores for extortionate strategists are 30% higher than generous strategists. Furthermore, half of the extortioners even earned scores higher than 3 per round on average, which is the score associated with constant mutual cooperation. By contrast, not a single generous strategist earned scores exceeding 3.

In the later rounds of the 500A extortionate treatment (500AE), the number of the extortionate strategists obtaining an average score exceeding 3 is significantly greater than those obtaining scores ≤3 (for further details see Supplementary Figs 1 and 3). In the last 60 rounds of 500AE, the average score of extortionate strategists rises to 3.127, which is significantly higher than 3 (*z*=2.301, *P*=0.021, Wilcoxon signed-rank test), while such a result was not observed in other conditions (for more details see Supplementary Fig 2 and Supplementary Tables 5, 6, 7, 8, 9 and 10). These indicate that extortion can indeed be a winning strategy under the conditions of awareness and long interaction length.

### Cooperation under extortionate and generous ZD strategies

On average, in the 500A, the cooperation rate of human co-players is 0.684±0.198 (mean±s.d.) in the extortionate treatment and 0.645±0.349 (mean±s.d.) in the generous treatment. There is no significant difference between the two treatments (Mann–Whitney's test, *n*_E_=*n*_G_=32, *z*=0.537, *P*=0.591). By contrast, there is a pronounced and statistically significant difference in human cooperation rates in favour of the generous strategy for the other treatments. The success of the generous strategy in the unawareness treatments (500U and 60U) supports the findings in Hilbe *et al*.^26^, while the result in the 60A treatment may be attributed to the limited learning experiences of human co-players. For details on average cooperation rates see Supplementary Table 1. For statistical results see Supplementary Table 11. For individual level data see Supplementary Tables 12 and 13.

**Figure 3:**
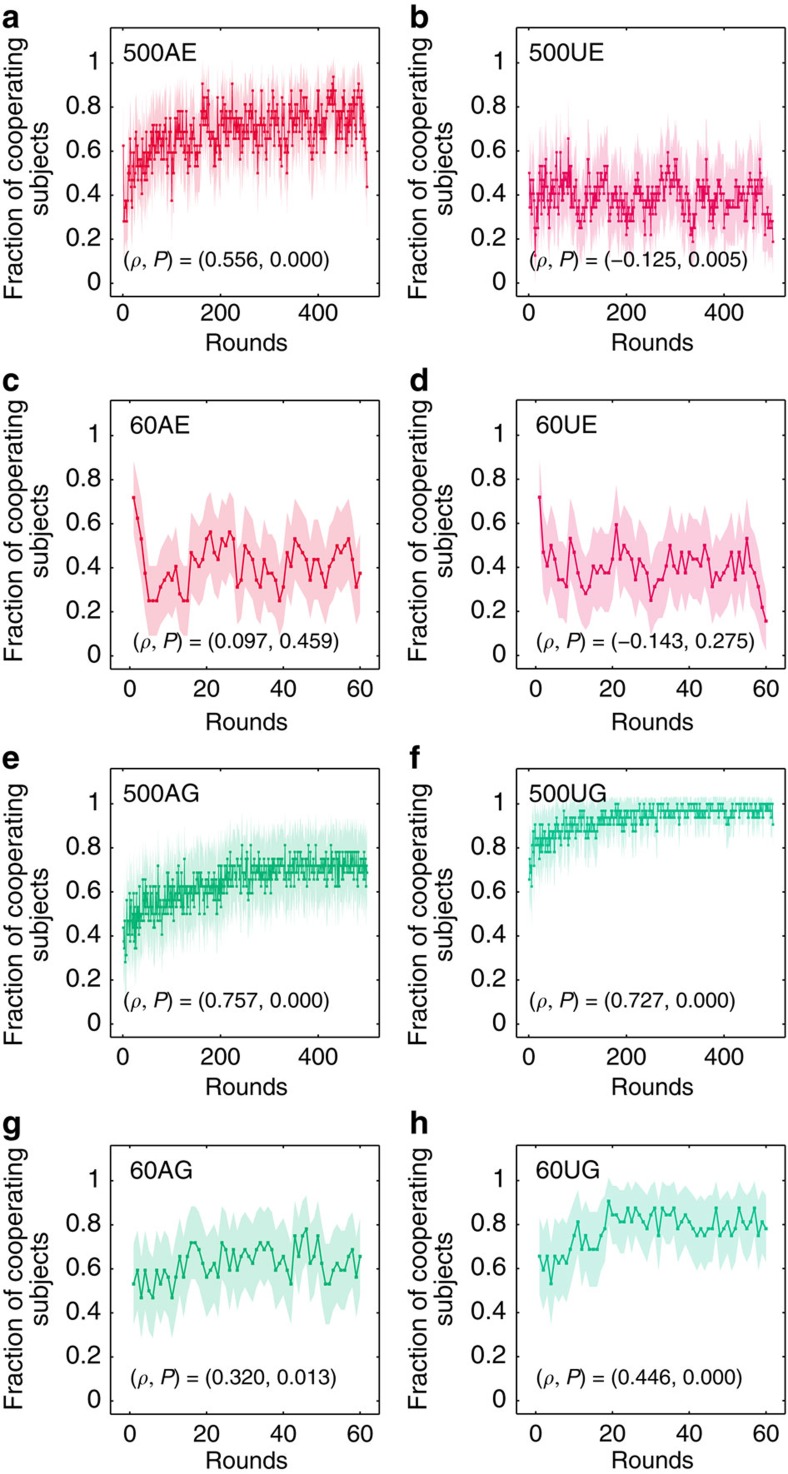
Human cooperation rates over the course of the game. The graph shows the fraction of cooperating human subjects for each round for extortionate treatment (**a**,**b**,**c**,**d**) and generous treatment (**e**,**f**,**g**,**h**). Dots represent the average human cooperation rate at a particular round within a treatment, with the shaded areas depicting the 95% confidence interval. The (colour) line indicates the time trend. Significant rising trends of cooperation behavior appear in both 500AE (**a**) and 500AG (**e**) treatments. ((*ρ*, *P*) in each subfigure indicates the Spearman's rank correlation coefficient and the corresponding *P* value). Significant positive time trends in cooperation were also found in the 500UG (**f**), 60AG (**g**) and 60UG (**h**) treatments, while there was no significant time trend in the 60AE (**c**) and 60UE (**d**) treatments. There was a significantly negative time trend in cooperation in the 500UE (**b**) treatment. Supplementary Figure 4 also provides the smoothed average time trends. In addition, Supplementary Figs 5 and 6 provide the smoothed time trend of individual human subjects.

Figure 3 shows the dynamic cooperation rate of human subjects for each treatment. In accordance with the similarity in cooperation rates across the 500-round awareness treatments (500AE and 500A generous treatment (500AG)), the two ZD strategies also generate similar upward dynamic patterns in these treatments (for Spearmans rank correlation see Fig. 3). The cooperation rate starts from a relatively lower point then follows an increasing path. For the 500AE, compared with the cooperation rate 0.563±0.221 (mean±s.d.) in the first 100 rounds, the cooperation rate 0.757±0.220 (mean±s.d.) in the last 100 rounds is significantly higher (Wilcoxon signed-rank test, *n*=32, *z*=4.207, *P*=0.000). For the 500AG, compared with the cooperation rate 0.513±0.327 (mean±s.d.) in the first 100 rounds, the cooperation rate 0.716±0.386 (mean±s.d.) in the last 100 rounds is also significantly higher (Wilcoxon signed-rank test, *n*=32, *z*=3.314, *P*=0.000). By contrast, in the 500U, 60A and 60U treatments, all generous ZD treatments displayed an upward trend in human cooperation, while the extortionate ZD treatments did not display this significant upward trend (for Spearmans rank correlation, see each subfigure in Fig. 3). The upward trend in human cooperation rates when playing against an extortionate ZD strategist, observed solely in the 500AE, can account for our main finding that extortion outperforms generosity in terms of scores, was realized only in the long length awareness treatment (500A) and not in the other treatments.

### The score relationship between ZD strategies and human players

**Figure 4:**
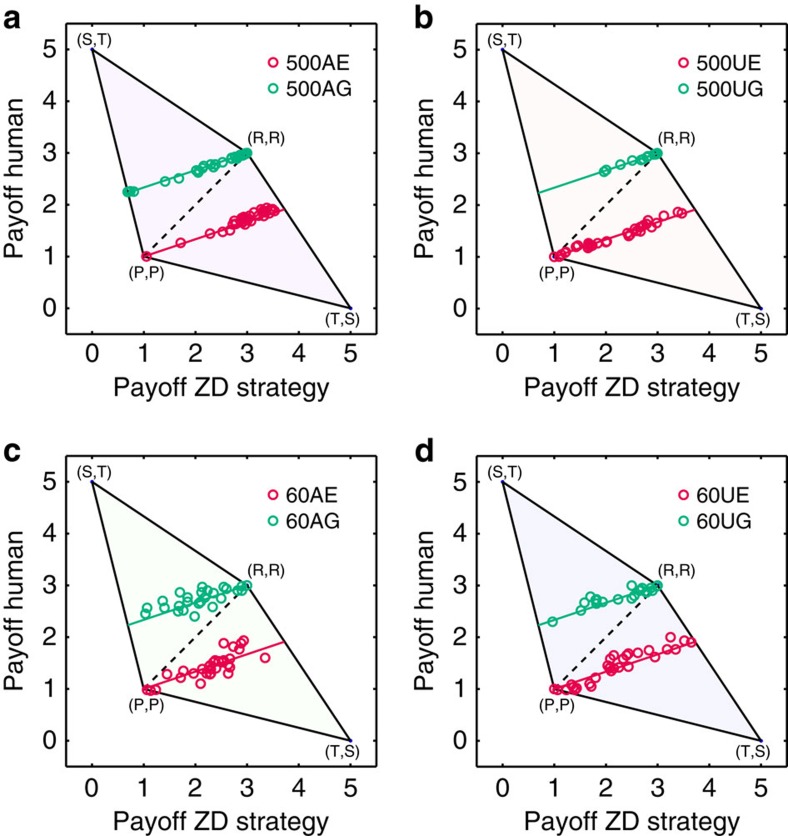
Experimental scores and theoretical prediction. For 500-round treatments (**a**,**b**) and 60-round treatments (**c**,**d**), the shaded area depicts the space of possible scores for the ZD strategy implemented by the ZD strategist (*x*-axis) and the human co-player (*y*-axis). The red (green) line corresponds to the theoretical prediction of the expected scores of extortion (generosity), respectively. For Extortion, the ZD strategist's score 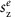 and his human co-player's score 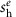 satisfy 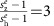 (red line), and the maximum scores for the ZD strategist and the human co-player are 3.727 and 1.907, respectively, while the minimum scores for both the ZD strategist and the human co-player are 1. For Generosity, the ZD strategist's score 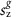 and his human co-player's score 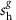 satisfy 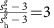 (green line), and the maximum scores for both the ZD strategist and the human co-player are 3, while the minimum scores for the ZD strategist and human co-player are 0.692 and 2.232, respectively. The open red (green) circles indicate the outcome of the extortionate (generous) treatment, respectively. Each circle indicates a pair of scores, the horizontal axis representing score of the ZD strategist and the vertical axis representing the human co-player's score. Corresponding to each line, there are 32 player pairs (circles) for each treatment (for individual level scores see Supplementary Tables 3 and 4).

The extortionate ZD strategist earns higher scores than their human co-players. On the contrary, the generous ZD strategists earn lower scores than their human co-players (as an illustration, see Fig. 2, for statistical results see Supplementary Table 14). The relationship of scores between ZD strategists and human co-players follows the linear relationship prediction in Press–Dyson^21^, as shown in Fig. 4. These results indicate that ZD strategies can indeed unilaterally enforce a linear relationship between human players' scores and their own scores.

## Discussion

The ZD strategies discovered by Press and Dyson^21^ allow a player in the IPD to unilaterally invoke a linear relationship between his own payoff and that of his opponent, by fixing a mixed strategy which conditions on the previous round's outcome. A remarkable feature of this class of strategies is that a ZD strategist can create a learning environment in which it is in fact optimal for his opponent to choose the cooperative action in each round. Two types of ZD strategies have attracted particular interest in the literature: so-called extortionate ZD strategy, in which the ZD strategist sometimes exploits the opponent's cooperation tendencies and never takes the initiative on cooperation, and the so-called generous ZD strategy, in which the opponent's cooperation tendency is never exploited, and sometimes cooperation is initiated by the ZD strategist. While both of these ZD strategies theoretically yield total cooperation from the opponent, the extortionate ZD strategy produces a clear score advantage for the ZD strategist^21^,^22^,^24^,^26^.

The prediction of the Press–Dyson^21^ theory gains support in our experiments. Our experiments showed that the extortionate strategy earned a higher score than the generous strategy, while continuously promoting the human co-player's cooperative behaviour at the same level of effectiveness as the generous strategy, when human subjects were aware of the nature of the opponent and had a sufficiently long experimental experience to learn. Under these conditions, after learning, the number of the successful extortionate strategists obtaining a higher score than mutual cooperation is significantly higher than those who obtain a score less than or equal to the score from mutual cooperation. We also found that the ZD strategies were successful in unilaterally enforcing a linear relationship between human players' scores and their own scores. These findings match the Press–Dyson^21^ theory well.

From an economics point of view, human subjects have no reason to reject a strategy which will provide a higher payoff unless there are some additional factors influencing their behaviours, assuming they have correctly found the optimal strategy. The fact that the cooperation rate reaches such a high level in the later stage of the 500AE treatment reflects individuals' pursuit of their own economic interests as well as their cognitive ability. However, human subjects' perception of the other player may be potentially responsible for the experimental result, for example, through fairness^36^,^37^,^38^,^39^ or conditional cooperation^40^,^41^,^42^. Hilbe *et al*.^26^ show that human subjects tended to be more cooperative when their opponent cooperated in the previous round. Human subjects in their study would defect when they played against the extortionate strategies, because the extortioner often defected. This conditional behaviour may even inhibit the process of discovering the profitable strategy. Therefore, the cooperation rate had difficulty increasing even in 500 unawareness extortionate (500UE) treatment. However, human subjects may be substantially less likely to demand fairness and reciprocity from a machine, compared with when they perceive that they are playing with another human being^36^,^37^,^38^,^39^. Through awareness, the performance of the strategy can be independent of potentially more complicated social influences such as human players' attitudes and intentions towards other players. The human subjects' cooperation rate given that the ZD strategist previously defected, is higher in the awareness condition than in the unawareness condition (see Supplementary Table 15). Consequently, the cooperation rates in the 500AE treatment reached a higher level. This indicates that awareness is one of the crucial factors for the Press-Dyson theory^21^ to hold in our experiment.

Another contributing factor could be the length of the experiment. As in Hilbe *et al*.^26^, our results also show that the extortionate ZD strategy cannot outperform the generous ZD strategy under a 60-round setting. This may be due to insufficient learning opportunities for the human subjects. A strategy involving uncertainty (and stochastic actions) takes a longer time to reveal itself than a pure strategy. The 60-round sessions implemented in the experiments may not be sufficient for humans to learn the probabilistic details of a ZD strategy. Lengthier sessions which can accommodate learning are often more ideal when subjects are facing probabilistic environments^43^,^44^,^45^,^46^,^47^,^48^,^49^. The ZD strategy is a probabilistic one, human subjects may need ample time in an evolutionary-like setting in order to learn the ZD strategy of his opponent, contemplate and formulate his own optimal strategy^50^. We observed that the human subjects' cooperation rates and the number of winning extortionists steadily increased in the 500AE treatment, reflecting the length of interaction effect, while this pattern did not appear in the other treatments.

In this research, we clarify that both factors, the knowledge of the opponent and the length of the interaction, have potential to facilitate the predictions of Press–Dyson^21^. However, we note that there may very well be other conditions in which the prediction of Press-Dyson theory^21^ can also be observed. Furthermore, our research leaves several related questions open, such as, whether playing against a computer is the same as playing against a human who uses a fixed strategy? Will knowing that the opponent's strategy is a fixed one shorten the time needed to find the profitable strategy? An additional observation from our experiment is that even with a lengthy period of time to learn, some players' cooperative behaviour remained unimproved. This may be due to either cognitive limitations of human subjects in realizing the profitability of cooperate strategy, or possible attitudes towards the game such that they deliberately refused to choose cooperate strategy. This points to some additional directions for future work to further check the robustness of ZD strategies' performances in various contexts, such as heterogeneous education levels of human subjects, across genders, or stochastic errors in the game^31^. We note also that in the first 60 rounds, the ZD strategists' scores and the human cooperation rates are different in the long-run and short-run treatments (see Supplementary Table 6). This might be due to the effect of the ‘shadow of future'^15^,^51^ or a ‘learning effect'^46^,^50^, which can be further studied in the future.

In real life, a prisoner's dilemma type setting can exist in many situations. For example, the interaction between a firm and its workers. Suppose that payoffs of the game are such that it is a dominant action in a one-shot interaction for workers to shirk instead of work diligently on the job, and it is a dominant action in a one-shot interaction for the firm to keep the worker's salary the same as before instead of giving a raise. The Press–Dyson^21^ theory implies that there are conditional randomized policies that the firm can implement which induces the worker to work diligently, some of which exploit the workers cooperative tendencies. Similar scenarios may exist between an online merchant and its consumers, or a developing country government and its citizens. As artificial intelligence products began to enter our daily life (for example, Siri, Robot, Self-driving cars, and so on), as an algorithm, the ZD strategies might also be applied by firms to maximize their profits. The experimental setting of unawareness, as in Hilbe *et al*.^26^ and our experiments, provides an ideal situation to test the interaction between individuals. However, the game can also be understood as the strategic interaction between countries, firms, or other institutions, or between institutions and human individuals. For an individual, the prospect of interacting with a machine-like opponent is not uncommon. For example, in the competition between a leader firm and a follower firm^52^ in a duopoly market, the leader firm can take a rigid policy towards the opponent, similar to a machine. Between the interaction of a state institution and a private entity, the institution may act like a machine^53^. In these two cases, the machine-like player's stature (leader firm or state institution) informs their opponents (follower firm or private sector). In this way, the ZD strategy also can be understood as an institutional (machine-like) strategy.

Although in the prisoner's dilemma, long-run relationships are typically regarded as a positive influence in preventing opportunistic behaviour while supporting cooperative behaviour^15^,^16^,^51^,^54^, now we can clearly see that this relationship can also be exploited by the Extortionate ZD strategist. Furthermore, since the extortionate strategy can obtain a higher payoff, as a potential mechanism, it will tend to be evolutionarily favored and reproduced. Our experimental results confirm that those policies designed with a ZD strategy in mind will have an evolutionary advantage by extorting rather than behaving generously under some conditions (for example, long-run interactions and awareness of a computer-like opponent). Against such a strategy, human subjects in this type of strategic setting cannot avoid being extorted unless they hold an outside option to exit the game altogether. Thus, these new strategies discovered by Press–Dyson^21^ raise serious challenges for the possibility of generous behaviour on the part of payoff maximizing policies towards actual human decision-makers.

## Methods

### Data source and experimental setting

The data was generated from our laboratory experiments which were conducted at The Experimental Social Science Laboratory of Zhejiang University. The experiments were implemented in accordance with standard social science experiment ethical guidelines and regulations.

Before the formal experiment, the subjects practiced with a matching pennies game against a computer to get acquainted with the laboratory setting. They were then assigned a set of materials including an instruction manual, an informed consent form and a recording chart for their use, and they played the game in a small isolated room with a computer. Oral instructions were also given. Subjects made decisions by clicking the option ‘C' or ‘D' on the screen. No communication was allowed during the experiment, and the subjects were asked to put their mobile phones in mute and sealed in an envelope until the end of the session. Considering the complexity of the ZD strategies, we provided human subjects with paper and pen to record their decision choices and scores round by round. In the awareness treatments, the human subjects were told that they would play a game with a fixed computer program. On the contrary, in the unawareness treatments, the human subjects were only told that they would play a game with a fixed opponent, which is similar to the implementation by Hilbe *et al*.^26^. The instruction manual of 500A is provided as an example in Supplementary Note 2. To obtain the desired number of rounds of data (500 or 60) for each subject while avoiding end-of-game effects^55^, human subjects were informed that the game would end with probability 0.1 in each round after the desired number of rounds (500 or 60) is reached.

During the experiments, the player earned scores according to the payoff matrix (see Fig. 1) and their choices. After the experiment, the sum of scores were converted to cash according to an exchange rate and paid to the subjects. For more details, see Supplementary Notes 1, 2 and 3.

### Statistical methods

Throughout the paper, we used the Mann–Whitney's test for the comparison between treatments, the Wilcoxon signed-rank test for the comparison within a treatment, and the Spearman's rank correlation test for detection of trends. In addition, we used the binomial probability test for comparison of the number of the Extortionate strategists who obtained an average score higher than the score from mutual cooperation, and the number of the extortionate strategists who obtained an average score less than or equal to the score from mutual cooperation.

## Additional information

**How to cite this article:** Wang, Z. *et al*. Extortion can outperform generosity in the iterated Prisoner's Dilemma. *Nat. Commun.* 7:11125 doi: 10.1038/ncomms11125 (2016).

## Supplementary Material

Supplementary Information Supplementary Figures 1-6, Supplementary Tables 1-15 and Supplementary Notes 1-3

## Figures and Tables

**Table 1:** Experimental design.

Treatment	Number of rounds	Information	ZD strategy	Number of human subjects
500AE	500	Awareness	Extortion	32
500AG	500	Awareness	Generosity	32
500UE	500	Unawareness	Extortion	32
500UG	500	Unawareness	Generosity	32
60AE	60	Awareness	Extortion	32
60AG	60	Awareness	Generosity	32
60UE	60	Unawareness	Extortion	32
60UG	60	Unawareness	Generosity	32

500AE, 500-round awareness extortionate treatment; 500AG, 500-round awareness generous treatment; 500UE, 500-round unawareness extortionate treatment; UG, 500-round unawareness generous treatment; ZD, zero-determinant strategy.
