# Supplementary material for: Extortion can outperform generosity in the iterated prisoner's dilemma
Source: Nat Commun. 2016 Apr 12;7:11125. doi: 10.1038/ncomms11125 (PMC4832060; doi:10.1038/ncomms11125)

1 Supplementary Figures

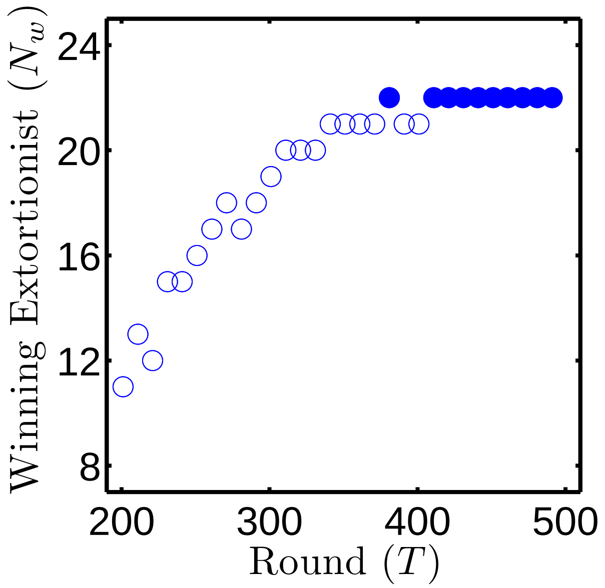

Supplementary Fig 1. Evolution of Number of Winning Extortionate ZD strategists (N_w) in 500AE treatment. With the payoff matrix specified in Figure 1, a score of 3 can be achieved by mutual cooperation, which is also the maximum score for a Generous ZD strategist. For each round T, define $S(T) :\equiv \frac{1}{200}\sum_{t=T-199}^{T} s(t)$ as the average scores over an interval (here the interval is 200 rounds, from round $T-199$ to T, and $s(t)$ is the score of the Extortionist at the round t). We define an Extortionist as a Winning Extortionist if $S(T) > 3$, otherwise, he is a Failed Extortionist. Circles represent the number of Winning Extortionists (N_w), and filled circles indicate that statistically, the number of Winning Extortionists is significantly greater than the number of Failed Extortionists. N_w significantly increases over time (Spearman's $\rho = 0.961$, p=0.000, n=301). When $T \geq 402$, the number of Winning Extortionists is greater than the number of Failed Extortionists ($p = 0.025$, binomial test, $n = 32$). This is evidence positively supporting the Press-Dyson prediction. **Supplementary table 10** provides the individual level data in last 200 rounds of the 500 round setting and **Supplementary Figure 3** also illustrates the average score comparison between different treatments in the last 200 rounds of the 500 round setting.

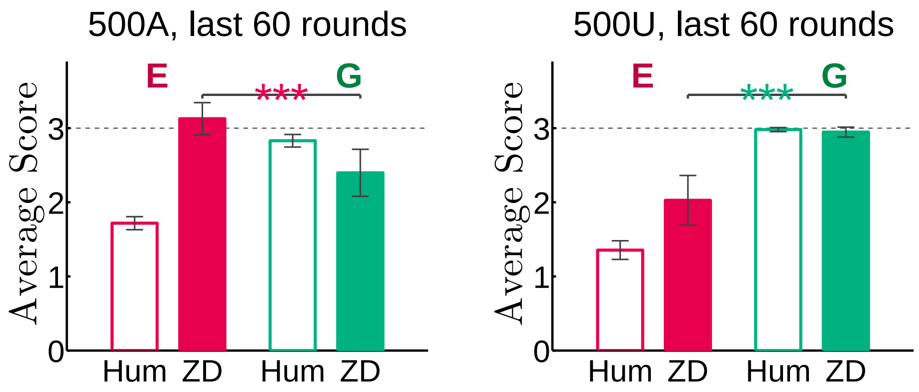

Supplementary Fig 2. Average scores of ZD strategists in the last 60 rounds in 500-round setting. The average score in the 500AE treatment during the last 60 rounds is significantly higher than in the 500AG treatment during the last 60 rounds ($z = 4.299$, $p = 0.000$, Mann-Whitney test, two-tailed). By contrast, the average score in 500UE during the last 60 rounds is significantly lower than in 500UG during the last 60 rounds ($z = -4.446$, $p = 0.000$, Mann-Whitney test, two-tailed). Three stars indicate $p < 0.001$. The error bars indicate the 95% confidence interval. In addition, the average score of Extortionate ZD strategists in the last 60 rounds in 500AE is 3.127, which is significantly larger than 3 ($z = 2.301$, $p = 0.021$, Wilcoxon signed-rank test). For the average ZD strategist's scores, see **Supplementary Table 6**, and for individual level data, see **Supplementary Table 8**.

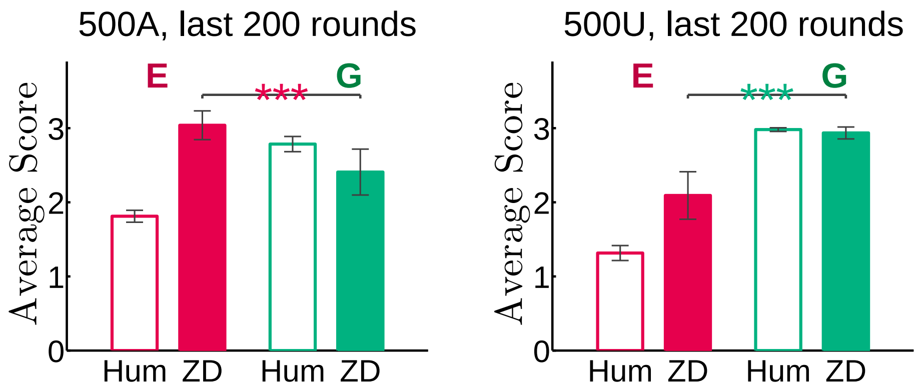

Supplementary Fig 3. Average scores of ZD strategists in the last 200 rounds in 500-round setting. The average score in the 500AE treatment during the last 200 rounds is significantly higher than in the 500AG treatment during the last 200 rounds ($z = 4.197$, $p = 0.000$, Mann-Whitney test, two-tailed). By contrast, the average score in the 500UE treatment during the last 200 rounds is significantly lower than in the 500UG treatment during the last 200 rounds ($z = -4.153$, $p = 0.000$, Mann-Whitney test, two-tailed). This indicates that the relationships between average Extortionate ZD and Generous ZD scores within the awareness and unawareness conditions were maintained until the end in the 500-round settings. Three stars indicate $p < 0.001$. The error bars indicate the 95% confidence interval. For the average ZD strategist's scores, see **Supplementary Table 9**, and for individual level data, see **Supplementary Table 10**.

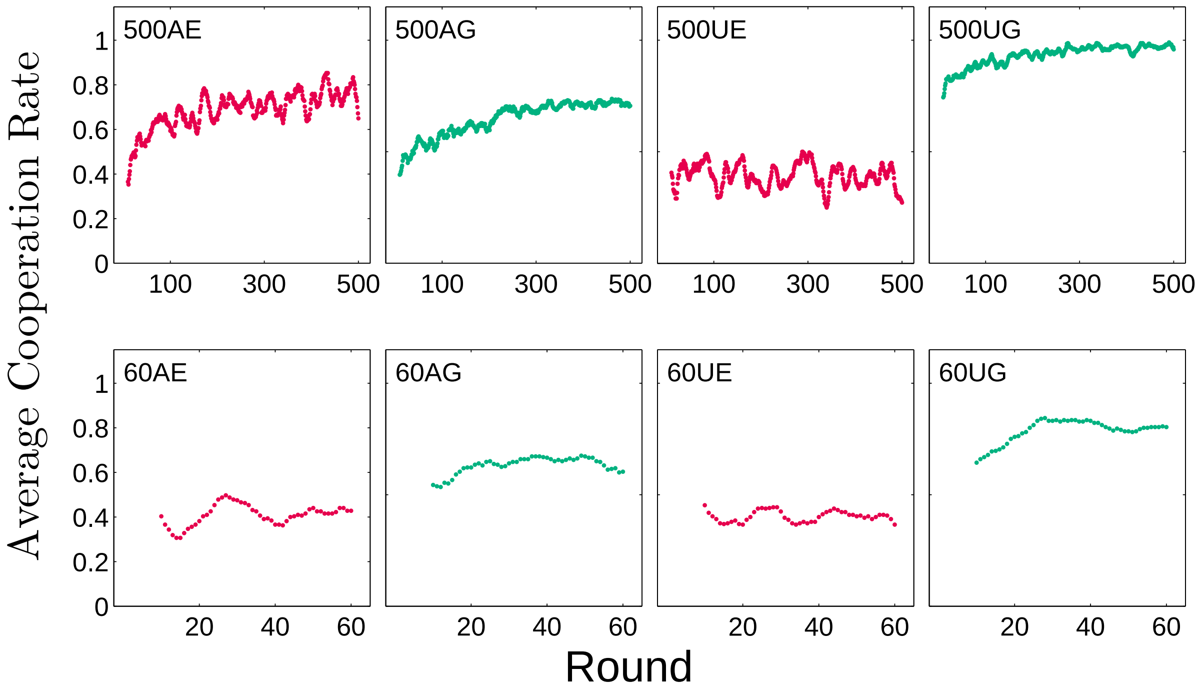

Supplementary Fig 4. Smoothed average cooperation rates. For each round T, define the Average Cooperation Rate $\bar{C}(T) :\equiv \frac{1}{10}\sum_{t=T-9}^{T}\bar{C}(t)$, where $\bar{C}_t = \frac{1}{32}\sum_{j=1}^{32} c_j(t)$, and $c_j(t)$ is the cooperation rate of subject j at round t.

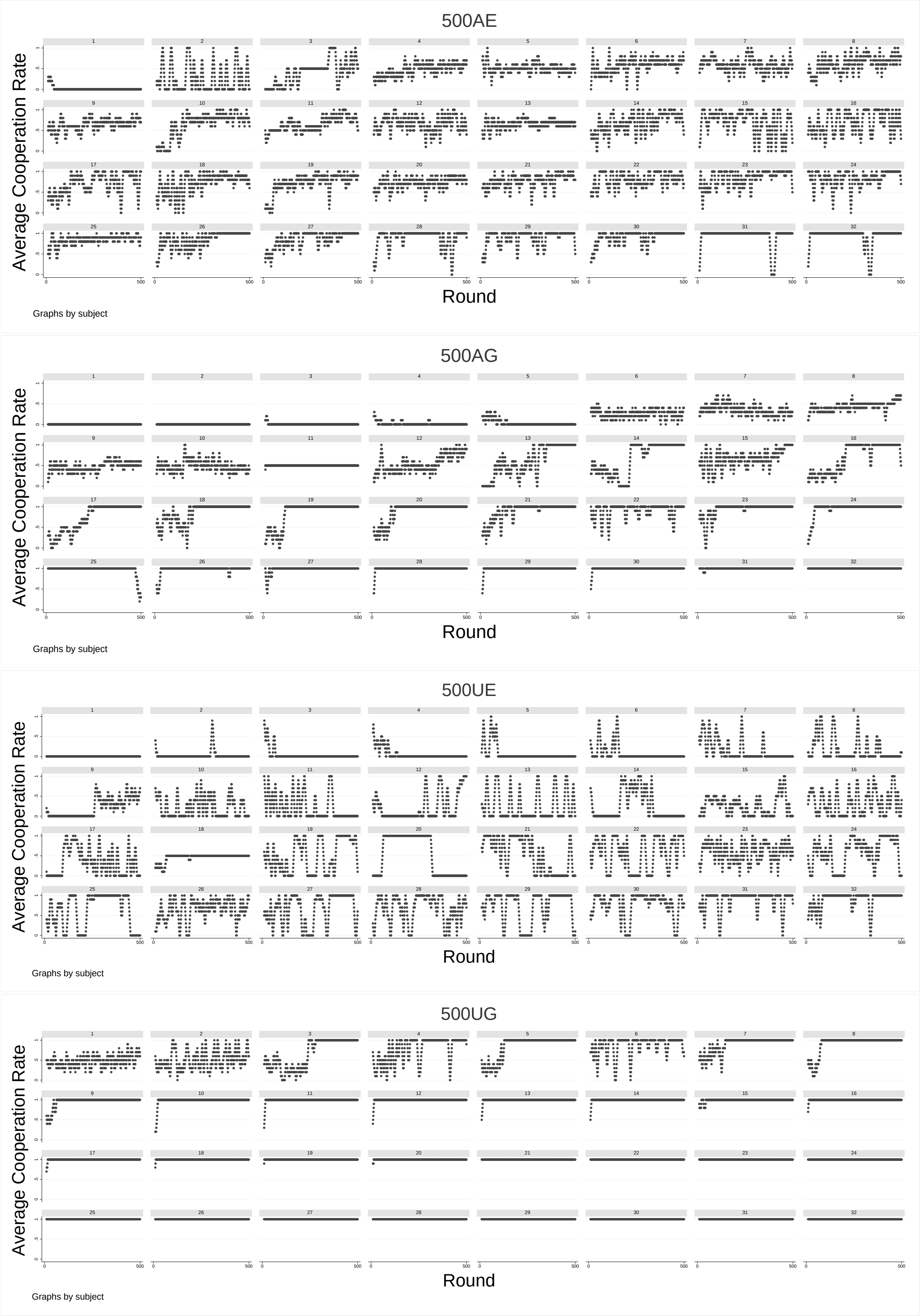

Supplementary Fig 5. Evolution of individual cooperation rates, 500-round treatments. For each round T, define the Average Cooperation Rate $C(T) :\equiv \frac{1}{10}\sum_{t=T-9}^{T} C(t)$, where $C(t)$ is the cooperation rate at round t.

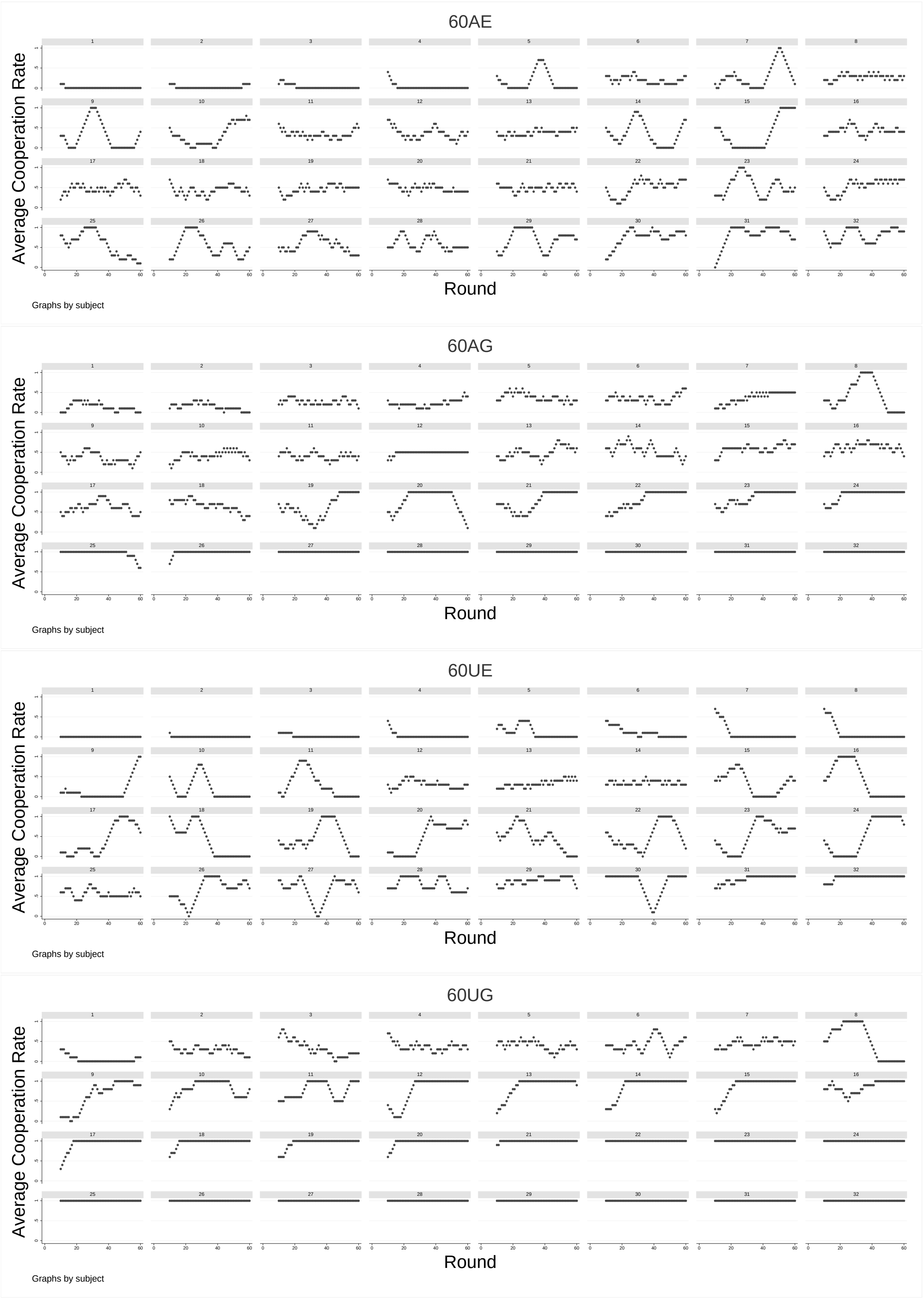

Supplementary Fig 6. Evolution of individual cooperation rates, 60-round treatments. For each round T, define the Average Cooperation Rate $C(T) :\equiv \frac{1}{10}\sum_{t=T-9}^{T} C(t)$, where $C(t)$ is the cooperation rate at round t.

2 Supplementary Tables

Treatment	Scores per round				Cooperation rate			
	Human		ZD strategist		Human		ZD strategist	
	mean	*s.d.*	*mean*	*s.d.*	*mean*	*s.d.*	*mean*	*s.d.*
500AE	1.703	0.200	2.943	0.511	0.684	0.198	0.436	0.137
500AG	2.746	0.260	2.263	0.788	0.645	0.349	0.741	0.245
500UE	1.371	0.253	2.115	0.726	0.390	0.264	0.242	0.170
500UG	2.953	0.093	2.860	0.281	0.931	0.138	0.950	0.100
60AE	1.419	0.263	2.247	0.564	0.416	0.204	0.250	0.143
60AG	2.788	0.189	2.252	0.604	0.624	0.285	0.732	0.199
60UE	1.416	0.320	2.158	0.737	0.402	0.275	0.254	0.191
60UG	2.862	0.170	2.536	0.576	0.776	0.270	0.841	0.187

Supplementary Table 1. Summary of the experimental results.

Condition	Comparison variables	z	p
500A	E vs. G	4.196	0.000
500U	E vs. G	−4.661	0.000
60A	E vs. G	0.034	0.973
60U	E vs. G	−2.349	0.019

Supplementary Table 2. Comparison of ZD strategists' scores within treatment. Mann-Whitney test results shown.

	500AE		500AG		500UE		500UG	
Group	Human	ZD	Human	ZD	Human	ZD	Human	ZD
1	1.002	1.052	2.248	0.688	1.000	1.000	2.644	1.964
2	1.264	1.714	2.248	0.688	1.002	1.102	2.678	2.008
3	1.440	2.280	2.252	0.702	1.040	1.140	2.782	2.282
4	1.476	2.526	2.260	0.740	1.078	1.208	2.860	2.510
5	1.508	2.668	2.256	0.806	1.082	1.212	2.872	2.682
6	1.620	2.720	2.450	1.410	1.094	1.224	2.886	2.726
7	1.630	2.770	2.510	1.680	1.192	1.422	2.924	2.794
8	1.690	2.750	2.622	2.052	1.218	1.458	2.942	2.812
9	1.620	2.960	2.658	2.068	1.198	1.698	2.976	2.926
10	1.630	2.780	2.664	2.014	1.182	1.652	2.978	2.958
11	1.736	2.956	2.728	2.348	1.172	1.652	2.982	2.972
12	1.654	2.904	2.706	2.136	1.232	1.662	2.992	2.972
13	1.732	3.002	2.750	2.160	1.262	1.662	2.990	2.980
14	1.744	2.894	2.778	2.298	1.218	1.738	2.990	2.980
15	1.682	2.922	2.778	2.378	1.262	1.842	2.988	2.988
16	1.764	2.934	2.776	2.386	1.296	2.016	3.000	2.990
17	1.698	3.008	2.822	2.522	1.282	2.002	2.998	2.988
18	1.726	2.996	2.888	2.698	1.500	2.600	3.002	2.992
19	1.684	3.074	2.906	2.696	1.402	2.442	2.998	2.998
20	1.788	3.048	2.924	2.764	1.458	2.418	2.998	2.998
21	1.850	3.130	2.892	2.802	1.428	2.468	3.000	3.000
22	1.790	3.220	2.928	2.818	1.542	2.562	3.000	3.000
23	1.782	3.322	2.960	2.860	1.576	2.666	3.000	3.000
24	1.910	3.270	2.954	2.914	1.490	2.670	3.000	3.000
25	1.880	3.330	2.964	2.934	1.610	2.730	3.000	3.000
26	1.876	3.306	2.984	2.954	1.634	2.784	3.000	3.000
27	1.852	3.352	2.982	2.972	1.572	2.882	3.000	3.000
28	1.836	3.426	2.994	2.974	1.704	2.814	3.000	3.000
29	1.938	3.378	2.988	2.978	1.652	2.982	3.000	3.000
30	1.910	3.450	2.990	2.980	1.792	3.122	3.000	3.000
31	1.914	3.494	2.998	2.998	1.864	3.394	3.000	3.000
32	1.880	3.530	3.000	3.000	1.840	3.470	3.000	3.000

Supplementary Table 3. Scores per round in the 500-round treatments.

	60AE		60AG		60UE		60UG	
Group	Human	ZD	Human	ZD	Human	ZD	Human	ZD
1	0.983	1.067	2.450	1.033	1.000	1.000	2.300	0.967
2	0.967	1.133	2.567	1.067	0.983	1.067	2.517	1.517
3	0.967	1.133	2.567	1.400	0.983	1.067	2.667	1.583
4	0.983	1.233	2.700	1.367	0.983	1.233	2.650	1.817
5	1.283	1.450	2.517	1.767	0.983	1.400	2.733	1.817
6	1.217	1.717	2.600	1.683	1.017	1.433	2.717	1.800
7	1.350	1.767	2.400	1.983	1.033	1.367	2.683	1.933
8	1.300	1.967	2.500	1.667	1.083	1.333	2.783	1.700
9	1.100	2.100	2.683	1.850	1.100	1.683	2.733	2.233
10	1.383	2.133	2.867	1.700	1.050	1.717	2.800	2.550
11	1.300	2.300	2.767	1.850	1.217	1.800	2.750	2.500
12	1.383	2.300	2.867	2.117	1.417	2.083	3.000	2.500
13	1.450	2.283	2.650	2.067	1.417	2.083	2.900	2.650
14	1.283	2.283	2.683	2.100	1.450	2.033	2.917	2.667
15	1.300	2.217	2.767	2.183	1.400	2.150	2.950	2.700
16	1.433	2.267	2.967	2.133	1.400	2.067	2.883	2.800
17	1.517	2.350	2.900	2.233	1.583	2.083	2.867	2.783
18	1.400	2.400	2.850	2.350	1.350	2.100	2.900	2.900
19	1.550	2.383	2.583	2.333	1.417	2.250	2.950	2.867
20	1.533	2.450	2.750	2.500	1.650	2.150	2.900	2.900
21	1.550	2.550	2.933	2.600	1.583	2.333	2.983	2.983
22	1.300	2.633	2.967	2.550	1.433	2.433	3.000	3.000
23	1.467	2.633	2.900	2.817	1.683	2.433	3.000	3.000
24	1.883	2.550	2.900	2.900	1.650	2.400	3.000	3.000
25	1.417	2.667	2.900	2.900	1.700	2.617	3.000	3.000
26	1.517	2.517	2.983	2.900	1.750	2.833	3.000	3.000
27	1.583	2.667	3.000	3.000	1.617	2.950	3.000	3.000
28	1.817	2.733	3.000	3.000	1.767	3.183	3.000	3.000
29	1.767	2.850	3.000	3.000	2.000	3.250	3.000	3.000
30	1.883	2.883	3.000	3.000	1.767	3.350	3.000	3.000
31	1.933	2.933	3.000	3.000	1.933	3.517	3.000	3.000
32	1.600	3.350	3.000	3.000	1.900	3.650	3.000	3.000

Supplementary Table 4. Scores per round in the 60-round treatments.

Treatment	Interval	N_w	N	Expected k	p (Binomial test)
500AE	first 60 rounds	3	32	16	0.000
500AG	first 60 rounds	0	32	16	0.000
500UE	first 60 rounds	5	32	16	0.000
500UG	first 60 rounds	0	32	16	0.000
500AE	last 60 rounds	22	32	16	0.025
500AG	last 60 rounds	0	32	16	0.000
500UE	last 60 rounds	5	32	16	0.000
500UG	last 60 rounds	0	32	16	0.000
60AE	all 60 rounds	1	32	16	0.000
60AG	all 60 rounds	0	32	16	0.000
60UE	all 60 rounds	5	32	16	0.000
60UG	all 60 rounds	0	32	16	0.000

Supplementary Table 5. Number of winning ZD strategists. In a given treatment (1st column), for each of the 32 ZD strategists, we calculate the average score (denoted by S) over an interval (2nd column). If the average score $S > 3$, a ZD strategist is called a winning strategist. The numbers of the winning strategists (denoted as N_w) observed in all treatments are shown in the 3rd column. In each treatment, there are in total N=32 ZD strategists samples. Denoting N_w as the number of observed successes and setting the null hypothesis that the probability of a success on a trial is 0.5 (or the expected k=16), we use a binomial probability test to report the statistical result (p-value of one-sided test) in the last column.

Treatment	Interval	Scores per round				Cooperation rate			
		Human		ZD strategist		Human		ZD strategist	
		mean	*s.d.*	*mean*	*s.d.*	*mean*	*s.d.*	*mean*	*s.d.*
500AE	first 60 rounds	1.597	0.285	2.480	0.557	0.514	0.217	0.338	0.164
500AG	first 60 rounds	2.719	0.193	1.922	0.742	0.490	0.319	0.649	0.210
500UE	first 60 rounds	1.409	0.293	2.167	0.657	0.399	0.243	0.247	0.170
500UG	first 60 rounds	2.884	0.156	2.649	0.496	0.826	0.241	0.872	0.172
500AE	last 60 rounds	1.718	0.243	3.127	0.603	0.747	0.230	0.465	0.159
500AG	last 60 rounds	2.830	0.236	2.398	0.879	0.718	0.385	0.804	0.258
500UE	last 60 rounds	1.355	0.349	2.027	0.930	0.365	0.337	0.231	0.221
500UG	last 60 rounds	2.981	0.076	2.947	0.187	0.972	0.098	0.979	0.076

Supplementary Table 6. Summary of results in the first 60 rounds and the last 60 rounds in the 500-round treatments.

Treatment	Comparison variables	z	p
500AE	last 60 rounds vs. first 60 rounds	4.376	0.000
500AG	last 60 rounds vs. first 60 rounds	3.395	0.001
500UE	last 60 rounds vs. first 60 rounds	−0.785	0.432
500UG	last 60 rounds vs. first 60 rounds	4.333	0.000

Supplementary Table 7. Comparison of scores of ZD strategists: the last 60 rounds vs first 60 rounds within treatment. Wilcoxon signed-rank test results shown.

	500AE		500AG		500UE		500UG	
Group	Human	ZD	Human	ZD	Human	ZD	Human	ZD
1	1.000	1.000	2.400	0.650	1.000	1.000	2.767	2.183
2	1.200	1.617	2.400	0.650	1.000	1.000	2.633	2.300
3	1.767	2.767	2.400	0.650	1.000	1.000	3.000	3.000
4	1.417	3.000	2.400	0.650	1.000	1.000	3.033	2.950
5	1.367	2.617	2.400	0.650	1.000	1.000	3.000	3.000
6	1.883	2.883	2.550	1.300	1.000	1.000	2.950	2.867
7	1.600	2.767	2.450	1.617	1.000	1.000	3.000	3.000
8	1.833	3.083	2.783	2.450	1.050	1.050	3.000	3.000
9	1.700	3.283	2.733	2.233	1.600	2.683	3.000	3.000
10	1.817	3.150	2.783	1.867	1.017	1.433	3.000	3.000
11	1.783	3.200	2.750	2.333	1.000	1.000	3.000	3.000
12	1.467	2.800	2.967	2.633	1.800	3.300	3.000	3.000
13	1.850	3.017	3.000	3.000	1.633	2.050	3.000	3.000
14	1.717	3.300	3.000	3.000	1.000	1.000	3.000	3.000
15	1.400	2.317	3.017	2.933	1.317	1.900	3.000	3.000
16	1.500	2.833	2.967	2.800	1.417	2.583	3.000	3.000
17	1.700	3.283	3.000	3.000	1.117	1.617	3.000	3.000
18	1.767	3.433	3.000	3.000	1.567	2.733	3.000	3.000
19	1.833	3.333	3.000	3.000	1.733	3.233	3.000	3.000
20	1.583	3.417	3.000	3.000	1.000	1.000	3.000	3.000
21	2.017	3.350	3.000	3.000	1.083	1.833	3.000	3.000
22	1.667	3.417	2.867	2.867	1.850	3.100	3.000	3.000
23	1.817	3.483	3.000	3.000	1.850	2.767	3.000	3.000
24	1.917	3.583	3.000	3.000	1.517	2.683	3.000	3.000
25	1.933	3.433	2.700	2.450	1.000	1.000	3.000	3.000
26	1.950	3.700	3.000	3.000	1.617	2.950	3.000	3.000
27	1.950	3.700	3.000	3.000	1.667	3.000	3.000	3.000
28	1.967	3.633	3.000	3.000	1.483	2.817	3.000	3.000
29	1.883	3.467	3.000	3.000	1.583	2.833	3.000	3.000
30	1.950	3.700	3.000	3.000	1.533	2.450	3.000	3.000
31	1.950	3.700	3.000	3.000	1.917	3.167	3.000	3.000
32	1.800	3.800	3.000	3.000	2.000	3.667	3.000	3.000

Supplementary Table 8. Average scores of the last 60 rounds in the 500-round treatments.

Treatment	Interval	Scores per round				Cooperation rate			
		Human		ZD strategist		Human		ZD strategist	
		mean	*s.d.*	*mean*	*s.d.*	*mean*	*s.d.*	*mean*	*s.d.*
500AE	last 200 rounds	1.812	0.223	3.039	0.538	0.740	0.211	0.494	0.150
500AG	last 200 rounds	2.785	0.284	2.408	0.860	0.713	0.383	0.788	0.271
500UE	last 200 rounds	1.315	0.280	2.092	0.889	0.376	0.315	0.220	0.194
500UG	last 200 rounds	2.981	0.068	2.936	0.223	0.968	0.109	0.978	0.078

Supplementary Table 9. Summary of results in the last 200 rounds in the 500-round treatments.

	500AE		500AG		500UE		500UG	
Group	Human	ZD	Human	ZD	Human	ZD	Human	ZD
1	1.000	1.000	2.260	0.685	1.000	1.000	2.705	2.030
2	1.350	1.675	2.260	0.685	1.020	1.145	2.740	2.165
3	1.710	2.760	2.260	0.685	1.000	1.000	3.000	3.000
4	1.540	2.765	2.260	0.685	1.000	1.000	2.985	2.860
5	1.585	2.610	2.260	0.685	1.000	1.000	3.000	3.000
6	1.765	2.915	2.355	1.480	1.000	1.000	2.960	2.885
7	1.575	2.600	2.450	1.550	1.000	1.100	3.000	3.000
8	1.805	2.980	2.640	2.315	1.100	1.225	3.000	3.000
9	1.790	3.115	2.730	2.280	1.395	2.370	3.000	3.000
10	1.955	3.180	2.625	1.975	1.115	1.490	3.000	3.000
11	1.930	3.205	2.755	2.330	1.080	1.330	3.000	3.000
12	1.670	2.795	2.770	2.495	1.375	2.175	3.000	3.000
13	1.880	3.030	2.975	2.975	1.330	1.680	3.000	3.000
14	1.920	3.220	2.990	2.990	1.005	1.230	3.000	3.000
15	1.645	2.495	2.770	2.645	1.265	1.890	3.000	3.000
16	1.855	3.055	2.970	2.895	1.390	2.215	3.000	3.000
17	1.805	3.130	3.000	3.000	1.075	1.750	3.000	3.000
18	1.895	3.345	3.000	3.000	1.500	2.750	3.000	3.000
19	1.885	3.285	3.000	3.000	1.550	2.850	3.000	3.000
20	1.825	3.200	3.000	3.000	1.015	1.215	3.000	3.000
21	1.920	3.270	2.995	2.995	0.995	1.545	3.000	3.000
22	1.910	3.285	2.905	2.880	1.575	2.725	3.000	3.000
23	1.955	3.530	3.000	3.000	1.560	2.760	3.000	3.000
24	2.045	3.370	3.000	3.000	1.565	2.940	3.000	3.000
25	2.040	3.390	2.910	2.835	1.525	2.900	3.000	3.000
26	2.065	3.615	2.985	2.985	1.595	3.045	3.000	3.000
27	2.050	3.475	3.000	3.000	1.655	3.280	3.000	3.000
28	1.830	3.305	3.000	3.000	1.510	2.835	3.000	3.000
29	2.040	3.440	3.000	3.000	1.660	3.260	3.000	3.000
30	1.985	3.560	3.000	3.000	1.635	3.235	3.000	3.000
31	1.915	3.290	3.000	3.000	1.795	3.420	3.000	3.000
32	1.835	3.360	3.000	3.000	1.805	3.580	3.000	3.000

Supplementary Table 10. Average scores of the last 200 rounds in the 500-round treatments.

Condition	Comparison variables	z	p
500A	E vs. G	-0.537	0.591
500U	E vs. G	-6.386	0.000
60A	E vs. G	-2.675	0.008
60U	E vs. G	-4.559	0.000

Supplementary Table 11. Comparison of human cooperation rates across treatments. Mann-Whitney test results shown.

	500AE		500AG		500UE		500UG	
Group	Human	ZD	Human	ZD	Human	ZD	Human	ZD
1	0.014	0.004	0.000	0.312	0.000	0.000	0.478	0.614
2	0.258	0.168	0.000	0.312	0.030	0.010	0.510	0.644
3	0.404	0.236	0.004	0.314	0.046	0.026	0.668	0.768
4	0.476	0.266	0.016	0.320	0.062	0.036	0.774	0.844
5	0.506	0.274	0.034	0.324	0.076	0.050	0.846	0.884
6	0.574	0.354	0.260	0.468	0.078	0.052	0.864	0.896
7	0.588	0.360	0.348	0.514	0.150	0.104	0.892	0.918
8	0.614	0.402	0.432	0.546	0.170	0.122	0.910	0.936
9	0.636	0.368	0.470	0.588	0.216	0.116	0.958	0.968
10	0.636	0.406	0.484	0.614	0.220	0.126	0.978	0.982
11	0.642	0.398	0.500	0.576	0.224	0.128	0.986	0.988
12	0.656	0.406	0.544	0.658	0.230	0.144	0.988	0.992
13	0.656	0.402	0.608	0.726	0.246	0.166	0.990	0.992
14	0.680	0.450	0.670	0.766	0.250	0.146	0.990	0.992
15	0.690	0.442	0.670	0.750	0.254	0.138	0.992	0.992
16	0.690	0.456	0.698	0.776	0.336	0.192	0.994	0.996
17	0.696	0.434	0.734	0.794	0.340	0.196	0.994	0.996
18	0.714	0.460	0.846	0.884	0.460	0.240	0.996	0.998
19	0.714	0.436	0.852	0.894	0.498	0.290	0.998	0.998
20	0.726	0.474	0.882	0.914	0.516	0.324	0.998	0.998
21	0.774	0.518	0.890	0.908	0.518	0.310	1.000	1.000
22	0.798	0.512	0.912	0.934	0.560	0.356	1.000	1.000
23	0.830	0.522	0.932	0.952	0.578	0.360	1.000	1.000
24	0.832	0.560	0.950	0.958	0.580	0.344	1.000	1.000
25	0.840	0.550	0.966	0.972	0.626	0.402	1.000	1.000
26	0.842	0.556	0.974	0.980	0.632	0.402	1.000	1.000
27	0.854	0.554	0.984	0.986	0.660	0.398	1.000	1.000
28	0.884	0.566	0.988	0.992	0.664	0.442	1.000	1.000
29	0.890	0.602	0.988	0.990	0.716	0.450	1.000	1.000
30	0.906	0.598	0.990	0.992	0.772	0.506	1.000	1.000
31	0.930	0.614	0.998	0.998	0.884	0.578	1.000	1.000
32	0.932	0.602	1.000	1.000	0.900	0.574	1.000	1.000

Supplementary Table 12. Individual Cooperation rates in the 500-round treatments.

	60AE		60AG		60UE		60UG	
Group	Human	ZD	Human	ZD	Human	ZD	Human	ZD
1	0.017	0.000	0.117	0.400	0.000	0.000	0.083	0.350
2	0.033	0.000	0.133	0.433	0.017	0.000	0.300	0.500
3	0.033	0.000	0.250	0.483	0.017	0.000	0.333	0.550
4	0.067	0.017	0.250	0.517	0.067	0.017	0.400	0.567
5	0.167	0.133	0.350	0.500	0.117	0.033	0.400	0.583
6	0.217	0.117	0.350	0.533	0.117	0.033	0.417	0.600
7	0.267	0.183	0.367	0.450	0.117	0.050	0.450	0.600
8	0.283	0.150	0.383	0.550	0.117	0.067	0.450	0.667
9	0.333	0.133	0.400	0.567	0.217	0.100	0.617	0.717
10	0.367	0.217	0.400	0.633	0.217	0.083	0.767	0.817
11	0.383	0.183	0.400	0.583	0.267	0.150	0.767	0.817
12	0.400	0.217	0.467	0.617	0.317	0.183	0.783	0.883
13	0.400	0.233	0.500	0.617	0.317	0.183	0.817	0.867
14	0.400	0.200	0.550	0.667	0.333	0.217	0.850	0.900
15	0.417	0.233	0.583	0.700	0.333	0.183	0.850	0.900
16	0.417	0.250	0.583	0.750	0.383	0.250	0.867	0.883
17	0.433	0.267	0.633	0.767	0.400	0.300	0.883	0.900
18	0.450	0.250	0.650	0.750	0.400	0.250	0.933	0.933
19	0.467	0.300	0.650	0.700	0.433	0.267	0.933	0.950
20	0.483	0.300	0.733	0.783	0.450	0.350	0.933	0.933
21	0.483	0.283	0.767	0.833	0.450	0.300	0.983	0.983
22	0.517	0.250	0.783	0.867	0.483	0.283	1.000	1.000
23	0.533	0.300	0.867	0.883	0.517	0.367	1.000	1.000
24	0.550	0.417	0.933	0.933	0.533	0.383	1.000	1.000
25	0.550	0.300	0.933	0.933	0.550	0.367	1.000	1.000
26	0.550	0.350	0.950	0.967	0.650	0.433	1.000	1.000
27	0.550	0.333	1.000	1.000	0.683	0.417	1.000	1.000
28	0.583	0.400	1.000	1.000	0.783	0.500	1.000	1.000
29	0.683	0.467	1.000	1.000	0.850	0.600	1.000	1.000
30	0.700	0.500	1.000	1.000	0.850	0.533	1.000	1.000
31	0.750	0.550	1.000	1.000	0.917	0.600	1.000	1.000
32	0.817	0.467	1.000	1.000	0.967	0.617	1.000	1.000

Supplementary Table 13. Individual Cooperation rates in the 60-round treatments.

Treatment	z	p
500AE	−4.937	0.000
500AG	4.910	0.000
500UE	−4.928	0.000
500UG	4.042	0.000
60AE	−4.938	0.000
60AG	4.642	0.000
60UE	−4.929	0.000
60UG	4.145	0.000

Supplementary Table 14. Score comparison between human subjects and ZD strategists within each pair, by treatment. Wilcoxon signed-rank test results shown.

Treatment	ZD cooperated in the previous round			ZD defected in the previous round		
	obs	*mean*	*s.d.*	*obs*	*mean*	*s.d.*
500AE	32	0.785	0.231	32	0.608	0.198
500AG	32	0.665	0.376	31	0.412	0.259
500UE	31	0.873	0.111	32	0.272	0.219
500UG	32	0.944	0.132	20	0.553	0.284
60AE	29	0.533	0.314	32	0.385	0.197
60AG	32	0.634	0.308	26	0.463	0.204
60UE	29	0.830	0.234	32	0.291	0.251
60UG	32	0.795	0.283	21	0.454	0.221

Supplementary Table 15. Human subjects' conditional cooperation rates.

3 Supplementary Notes

Supplementary Note 1: Experimental Methods

We recruited 256 volunteers including undergraduate and graduate students from different majors at Zhejiang University. These volunteers participated in 64 groups of four subjects each in a computerized experiment. The 500EA and 500GA treatments were conducted on November 19 and 21, 2014. Each session lasted about 60 minutes in total. The 500UE and 500UG treatments were conducted on October 17 and 18, 2015. Each session lasted about 75 minutes in total. The 60AE, 60AG, 60UE and 60UG treatments were conducted between October 29 and November 22, 2015. Each session lasted about 20 minutes in total. Human subjects earned an average of 50 Yuan RMB (Chinese Yuan, about USD 7.87) for the 500-round settings and 11 Yuan RMB (about USD 1.73) for the 60-round settings. For reference, the typical student assistant's wage at Zhejiang University is from 15 to 25 Yuan RMB (about USD 2.36 to 3.94) per hour.

Before the experiment began, an experimenter verbally informed every subject about how to operate the computer, and that all the earnings they would receive would be paid in cash. To ensure the subjects' anonymity, subjects would have to make their decisions under a neutral pseudonym and were not allowed to talk to each other during or after the experiment. When the experiment was completed, subjects would receive their payment anonymously, as the procedure was conducted in a separated room.

Subjects were randomly assigned to a computer workstation, which was separated by opaque partitions between each workstation. Once seated, subjects received the instructions to learn about the rules of the game. There was no time limit for each decision.

The experiment was conducted using a computer program designed by author Zhijian Wang to run laboratory games.

Supplementary Note 2: Instructions of the experiment (example)

Taking the 500AE treatment as an example, the printed instructions provided to subjects (translated from Chinese) are provided below:

You are participating in a social science experiment. Your earnings will be determined by your decisions and the decisions of your computer partner. It is very important for you to read these instructions carefully.

This instruction booklet is the only information which will be provided to you during the experiment. No communication with other participants is allowed during the experiment. Please raise your hand if you have any questions.

Virtual points are the currency used in this experiment. All of your gains are calculated in terms of virtual points. When the experiment ends, the virtual points you earn will be exchanged to yuan RMB (CNY). In addition, there is a 5 yuan show-up payment. So when the experiment ends, you will get:

Your Cumulative Virtual Points*0.04+5 (show up payment) yuan RMB (CNY)

The earning will be paid to you in cash.

As the experiment begins, the computer program will assign you to a computer partner. After 500 rounds of play, the experiment will end with probability 0.1 in each subsequent round. In each round, you and your computer partner will choose a letter (C or D). Both of you will make your decisions without knowing the other's decision. Your earning is determined by your decision and that of your partner. The following table shows all the possible earnings in each round. The first number in each cell is your earnings, while the second number is your partner's:

		Your Partner's Decision			
		C		D	
Your	C	3	3	0	5
Decision	D	5	0	1	1

- You: C, Your partner: C   You will receive 3, Your partner will receive 3
- You: C, Your partner: D   You will receive 0, Your partner will receive 5
- You: D, Your partner: C   You will receive 5, Your partner will receive 0
- You: D, Your partner: D   You will receive 1, Your partner will receive 1

As the experiment begins, the following information and input window will be on your computer screen:

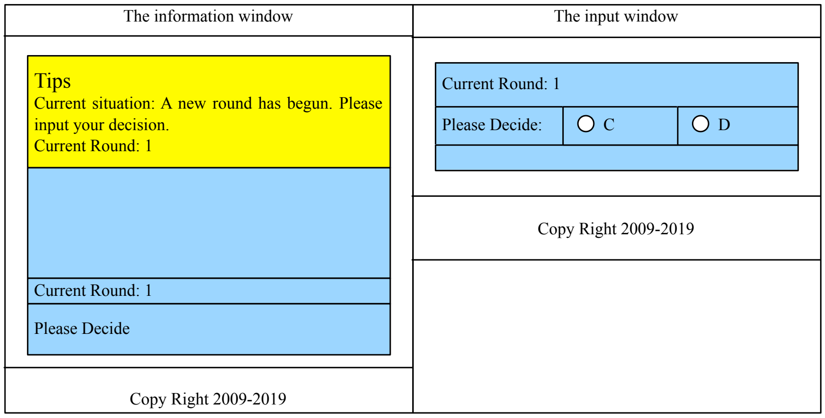

The window is divided into two parts. On the left is the information window while on the right is the input window. In the left information window, you will observe information about the current round. In the right input window, it lists the options C and D, which can be chosen by using the mouse to click on the white circle before C or D.

After one round, you will get observe following information and input window:

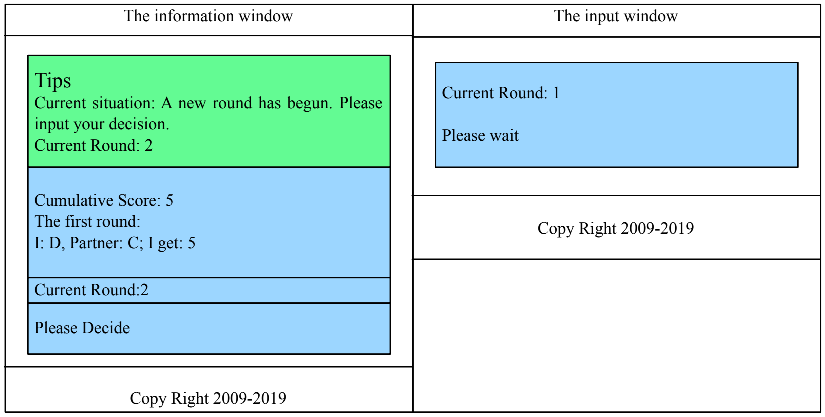

In the information window, the information about your decisions, your partner's decisions, and your scores is displayed. Please record your decisions and scores on the paper table provided to you. When you are ready, use F5 to refresh. Then the right input window will list your options C and D (as below):

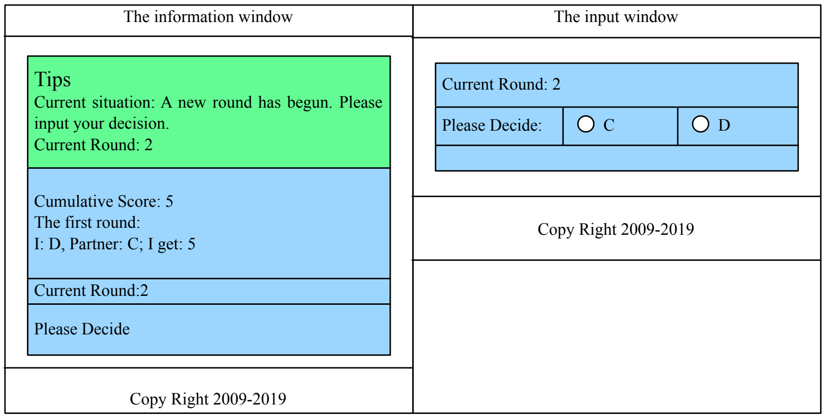

Click the letter you want to choose in this round.

The information window also provides your cumulative score. When you finish your experiment, please record the cumulative round and cumulative score.

Now, the experiment will begin.

Supplementary Note 3: Screenshots of the experiment

As the experiment begins, the following information and input window will be on the computer screen:

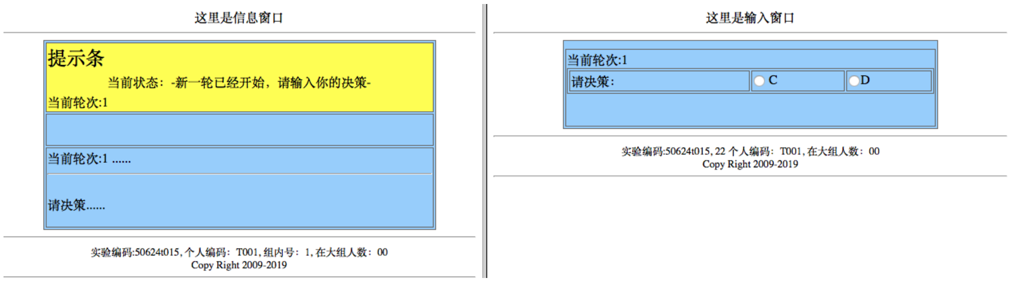

After one round, you will observe the following information window and input window:

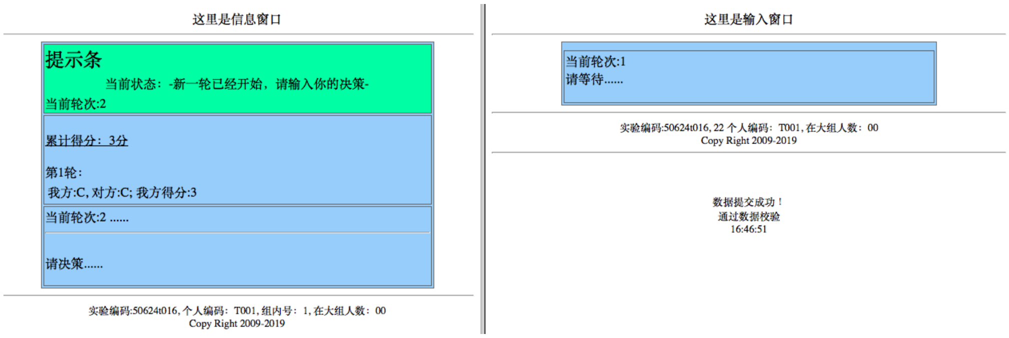

Using F5 to refresh, the right input window will then list your options C and D (as below):

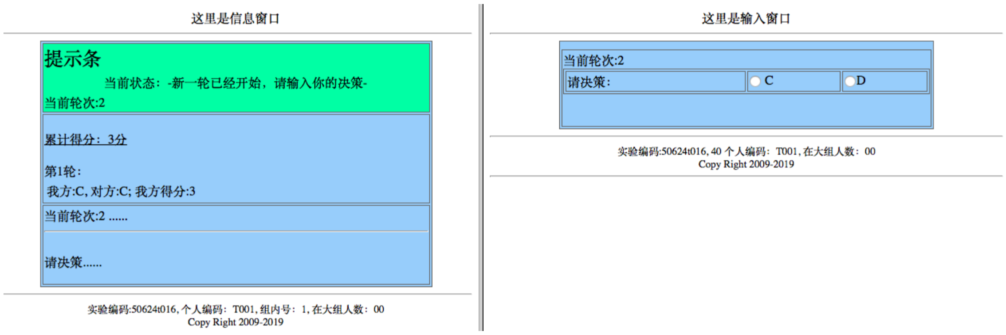

Supplement: Supplementary Information — Supplementary Figures 1-6, Supplementary Tables 1-15 and Supplementary Notes 1-3 [file ncomms11125-s1.pdf]
